# Structural Insights into the Autoregulation and Cooperativity of the Human Transcription Factor Ets-2*

**DOI:** 10.1074/jbc.M114.619270

**Published:** 2015-02-10

**Authors:** Joseph A Newman, Christopher D. O. Cooper, Hazel Aitkenhead, Opher Gileadi

**Affiliations:** From the Structural Genomics Consortium, University of Oxford, Oxford OX3 7DQ, United Kingdom

**Keywords:** Allosteric Regulation, Cellular Senescence, DNA Binding Protein, ETS Transcription Factor Family, Transcription Factor, X-ray Crystallography

## Abstract

Ets-2, like its closely related homologue Ets-1, is a member of the Ets family of DNA binding transcription factors. Both proteins are subject to multiple levels of regulation of their DNA binding and transactivation properties. One such regulatory mechanism is the presence of an autoinhibitory module, which in Ets-1 allosterically inhibits the DNA binding activity. This inhibition can be relieved by interaction with protein partners or cooperative binding to closely separated Ets binding sites in a palindromic arrangement. In this study we describe the 2.5 Å resolution crystal structure of a DNA complex of the Ets-2 Ets domain. The Ets domain crystallized with two distinct species in the asymmetric unit, which closely resemble the autoinhibited and DNA bound forms of Ets-1. This discovery prompted us to re-evaluate the current model for the autoinhibitory mechanism and the structural basis for cooperative DNA binding. In contrast to Ets-1, in which the autoinhibition is caused by a combination of allosteric and steric mechanisms, we were unable to find clear evidence for the allosteric mechanism in Ets-2. We also demonstrated two possibly distinct types of cooperative binding to substrates with Ets binding motifs separated by four and six base pairs and suggest possible molecular mechanisms for this behavior.

## Teaser

**Background:** ETS transcription factors regulate expression of genes involved in development and cancer.

**Results:** Crystal structure of the Ets-2 DNA binding domain reveals how closely spaced binding sites are recognized.

**Conclusion:** A significant difference in binding regulation is observed between the closely related Ets-1 and Ets-2 transcription factors.

**Significance:** This may explain the different physiological roles of different Ets proteins.

## Introduction

The Ets^4^ (E26 transformation-specific) family of transcription factors are conserved throughout the metazoan phyla (1) with 28 members in the human genome (2). Ets proteins share an 85-amino acid winged helix-turn-helix DNA binding domain (the Ets domain) that recognizes the conserved core DNA sequence GGA(A/T) through direct polar contacts mediated by residues in the highly conserved recognition helix (α3). Ets family proteins have been assigned into four classes based on the binding preferences of up to two residues upstream and downstream of the GGA(A/T) core (3). Class I proteins (which include Ets-2) are by far the most widely represented, comprising more than half of the human Ets proteins. Despite the similarities in the mode and sequence specificity of DNA binding, the functional roles and biological processes regulated by this family of transcription factors are diverse, being able to both activate and repress the transcription of genes involved in proliferation, differentiation, and apoptosis of various cell types. Unsurprisingly, given their importance in cellular proliferation and cell death, a number of Ets family members, (*e.g.* Ets-1, Pu-1, Etv-1, Etv-4, and Tel) are associated with tumorigenesis and tumor progression. Various factors influence the functional roles of Ets proteins, including tissue-specific expression, alternate splicing, subcellular localization, presence of additional domains, post translational modifications, and the modulation of DNA binding through the formation of homomeric or heteromeric interactions with protein partners.

Several of the Ets family members such as Ets-1, Ets-2, Etv-4, Etv-5, and Etv-6 are subject to autoinhibition in which additional structural elements outside of the core Ets domain inhibit DNA binding. Much of the knowledge of autoinhibition in Ets family proteins comes from studies on Ets-1, which is closely related to Ets-2 (84% sequence identity for the Ets domains and 55% across the entire length of the polypeptide) and, like Ets-2, contains 4 additional α-helices located immediately N-terminal (HI-1 and HI-2) and C-terminal (H4 and H5) to the core Ets domain. Deletion of these helices or expression of an alternately spliced isoform of Ets-1, which lacks these elements, results in a protein with increased DNA binding affinity (4–6). Structural and functional studies have established a mechanism for the autoinhibition of Ets-1 where, in the absence of DNA, the additional helices (HI-1 HI-2 and H4 H5) form a four-helix bundle that serves as an allosteric inhibitory module, packing against the opposite face from the DNA binding interface (7). Upon binding DNA the first N-terminal inhibitory helix HI-1 unfolds (8, 9), forming a mostly disordered section of random coil (7). The effect of this allosteric inhibition in Ets-1 is increased by phosphorylation of serine residues immediately N-terminal to HI-1 (10) and can be relieved by protein-protein interactions (11, 12). A further layer of complexity was added to this model when it was discovered that the presence and phosphorylation status of an intrinsically disordered serine-rich region immediately N-terminal to HI-1 was found to stabilize the inactive state of Ets-1 to a much greater extent (10–20-fold inhibition) than the structural transitions of HI-1 (2–3-fold inhibition) (13, 14). A recent study established a possible mechanism by which this inhibition proceeds, finding that the phosphorylated serine-rich region is intrinsically disordered and forms dynamic interactions with the core Ets domain, including regions of the DNA recognition interface (15).

Another mechanism of regulation that is shared by both Ets-1 and Ets-2 is the phenomenon of cooperative binding to palindromic repeats of the Ets binding site (EBS) (16–18), which is not generally observed in the wider Ets family proteins, which typically bind single EBS motifs with high affinity. Structural studies on Ets-1 bound to a palindromic EBS repeat in the core GGA(A/T) motif separated by 4 bp (a sequence naturally occurring on the stromelysin-1 promoter) have identified a small predominantly polar interface between monomers that may explain the structural basis of cooperative binding (19, 20). Although the contact area for this interface is minimal, it is formed from residues present in the N-terminal autoinhibitory regions, and the stimulation of binding to cooperative sites was found to be approximately equal to the relief of autoinhibition (11, 19). An intriguing aspect of the structural studies of Ets-1 is the propensity for the dynamic HI-1 inhibitory helix to associate with a neighboring Ets-1 molecule in a domain-swapped manner. This phenomenon has been observed for both free (PDB IDs 1MD0 (12) and 1GVJ) and DNA-bound Ets-1 structures (PDB ID 3RI4 (21), 2NNY (19), and 3MFK (20)). In one of these structures (PDB ID 2NNY (19)) HI-1 was modeled as a random coil; however, a re-examination of the electron density maps reveals clear density for a domain-swapped helix similar to that observed in PDB ID 3MFK (20). It is not clear if this propensity for domain swapping has any functional significance, yet the fact that it is present in multiple structures with different crystal forms and involves the same regions that have been shown to be important for allosteric transitions suggest that it may have a functional importance for relief from autoinhibition or cooperativity.

Despite the many similarities between Ets-1 and Ets-2 in their structural and regulatory mechanisms, the function of the two paralogues *in vivo* is distinctly different. It is clear that some of these differences can be explained by the fact that they have different tissue-specific expression profiles, with both proteins expressed in a wide range of cell types, but Ets-1 is much more highly expressed in thymus, lung, and Jurkat cells, with corresponding differences in the phenotypes of knock-out mice (22). Nevertheless, significant differences have been observed in their transactivation activity and the protein-protein interactions in which they participate; for example, Ets-2 but not Ets-1 is able to interact with both *ERG* (23) and *CDK10* (24). To gain insights into the structural basis for these functional differences and to further investigate the structural basis for autoinhibition and cooperativity, we have determined the crystal structure of the Ets domain of Ets-2. The structure, determined in complex with a single EBS DNA oligonucleotide, unexpectedly contains archetypes of both the autoinhibited and non autoinhibited forms, allowing us to analyze the structural and conformation differences that accompany autoinhibition. The association and interfaces formed between molecules in the asymmetric units give insights into novel aspects of the autoinhibitory mechanism and cooperative binding.

## EXPERIMENTAL PROCEDURES

### 

#### 

##### Cloning and Overexpression

Plasmid DNA templates for full-length human Ets-2 (IMAGE: 3852274) and full-length murine Ets-1 (IMAGE: 40056547) were obtained from the Mammalian Gene Collection (Source BioScience, Nottingham, UK).

The fragments of Ets-2 and Ets-1 used in this study are: Ets-2^325–464^ (Ets and autoinhibition domains, crystallized fragment), Ets-2^308–469^ (Ets domain, autoinhibition domain and N-terminal flanking sequence, used in electrophoretic mobility shift assays (EMSAs)), Ets-2^325–469^ (Ets and autoinhibition domains, used in EMSA), Ets-2^360–464^ (ETS domain, used in EMSA), Ets-1^280–440^ (Ets domain, autoinhibition domain, and N-terminal flanking sequence, used in EMSA), Ets-1^300–440^ (Ets and autoinhibition domains, used in EMSA), Ets-1^331–440^ (Ets domain, used in EMSA). The gene fragments were amplified by PCR and cloned into the pNIC28-Bsa4 expression vector, encompassing a tobacco etch virus protease-cleavable N-terminal His tag MHHHHHHSSGVDLGTENLYFQ↓SM), as described elsewhere (25). Plasmids were transformed into BL21(DE3)-R3-pRARE2, and cultures were grown in UltraYield baffled flasks (Thomson Instrument Co.) in Terrific Broth medium containing 50 μg/ml kanamycin at 37 °C to an *A*_600_ of 2–3, at which point the cultures were cooled to 18 °C and expression was induced by the addition of 0.1 mm isopropyl β-d-1-thiogalactopyranoside, with cells harvested 18 h after induction.

##### Protein Purification

For purification of both the Ets-1 and Ets-2 domains, cell pellets were thawed and resuspended in buffer A (50 mm HEPES, pH 7.5, 500 mm NaCl, 5% glycerol, 10 mm imidazole, 0.5 mm Tris(2-carboxyethyl)phosphine) and disrupted by sonication. Cell debris and nucleic acids were removed by the addition of 0.15% polyethyleneimine, pH 7.5, and centrifugation at 50,000 × *g* for 1 h at 4 °C. The supernatants were applied to a 3-ml Ni^2+^-iminodiacetic acid (Ni-IDA) agarose immobilized metal ion affinity chromatography gravity flow column, washed with wash buffer (buffer A with 30 mm imidazole), and eluted with 5 column volumes of elution buffer (buffer A with 300 mm imidazole). Proteins were incubated overnight at 4 °C in the presence of tobacco etch virus protease (1:40 mass ratio) while being dialyzed using 3.5-kDa molecular weight cutoff snakeskin membrane (Thermo Fisher Scientific, Rockford, IL) into buffer B (20 mm HEPES, pH 7.5, 500 mm NaCl, 5% glycerol, 0.5 mm Tris(2-carboxyethyl)phosphine). Tobacco etch virus protease and contaminating proteins were removed by reapplication of dialyzed proteins to a Ni^2+^-iminodiacetic acid agarose immobilized metal ion affinity chromatography column (2-ml column volume). Proteins passing through the column were pooled and concentrated using a 10-kDa molecular weight cutoff centrifugal concentrator to 1 ml before loading onto a HiLoad 16/60 Superdex S75 gel filtration column equilibrated in buffer B. Proteins were identified by SDS-PAGE and confirmed by mass spectrometry, and concentrations were determined by absorbance measurement at 280 nm (Nanodrop) using the calculated molecular mass and extinction coefficients.

##### Crystallization and Structure Determination

For crystallization of the Ets domain DNA complex, the oligonucleotides ACCGGAAGTG and CACTTCCGGT were resuspended to 900 μm in 10 mm Tris-HCl, pH 8.0, 50 mm NaCl, mixed in a 1:1 ratio, heated to 95 °C for 5 min in a heating block, and allowed to cool slowly over several hours. The Ets-2 Ets domain protein was concentrated to 300 μm (5 mg/ml) and mixed with an equal volume of double-stranded DNA (1:1.5 molar ratio) before being concentrated on a 3-kDa molecular weight cutoff centrifugal concentrator to 15 mg/ml for crystallization. Sitting drop vapor diffusion crystallization trials were set up with a Mosquito (TTP Labtech) crystallization robot. Crystals grew at 20 °C from conditions containing 0.1 m BisTris, pH 5.5, 0.25 m NaCl, and 15% PEG 3350 and were transferred to a cryoprotectant solution consisting of well solution supplemented with 25% ethylene glycol before being loop-mounted and plunged into a pool of liquid nitrogen. Diffraction data were collected at Diamond Light Source beamline I02 and processed using XDS (26), and the structure was solved by molecular replacement using the program MOLREP (27) with the structure of the FEV DNA complex (PDB ID 3ZP5) as a search model. Model building was performed using the program COOT (28) and refined using and PHENIX REFINE (29). A summary of the data collection and refinement statistics is found in Table 1.

##### Electrophoretic Mobility Shift Assays

The affinity and cooperativity of Ets-2 Ets domain DNA binding was measured using EMSAs. The probes consisted of the following oligonucleotide sequences annealed to the complementary strands (the core Ets binding sites are underlined): single site (ATCTCACC**GGAA**GTGTAGCA) and palindromes (4-bp, AGC**GGAA**GTAC**TTCC**GGA; 5-bp, AGC**GGAA**GTGAC**TTCC**GGA; 6-bp, AGC**GGAA**GTGCAC**TTCC**GGA; 7-bp, AGC**GGAA**GTGACAC**TTCC**GGA; 8-bp, AGC**GGAA**GTGATCAC**TTCC**GGA).

Radiolabeled double-stranded DNA probes were prepared by incubating the forward strand oligonucleotides for 1 h at 37 °C with T4 polynucleotide kinase in the presence of [γ-^32^P]ATP. Complementary (non-radiolabeled) oligonucleotides were added, and the mixture was heated to 95 °C and allowed to cool slowly to room temperature. The double-stranded DNA probes were purified before use on a Bio-Rad P6 micro-biospin column. EMSAs were performed by incubating radiolabeled probe (at a concentration of 0.1 nm for Ets-2 and 0.02 nm for Ets-1) with protein titrated by serial dilution. The buffer was 50 mm Tris-HCl, pH 7.5, 25 mm NaCl, 50 mm l-arginine-HCl, pH 7.5, 0.5 mm EDTA, 0.1% Tween 20, 2 mm DTT, and 5% glycerol (inclusion of arginine in the buffer prevented the precipitation of protein-DNA complexes). Reactions were performed for 15 min at room temperature, and 5 μl of each reaction was mixed with loading dye to 0.25% and resolved by 12% native polyacrylamide gel electrophoresis in Tris borate EDTA buffer at 180 V for 1 h on ice. Gels were visualized using phosphorimaging, quantitation was performed using quantity one 1-D analysis software (Bio-Rad), and apparent dissociation constants were calculated using a sigmoidal four-parameter logistic nonlinear regression model in PRISM (GraphPad).

##### Modeling of Ets-2 on Palindromic DNA Substrates

To assess potential interactions between Ets-2 molecules on the various palindromic DNA substrates, we have superposed chain A of the Ets-2 structure as a rigid body onto successive positions on the pseudo-contiguous DNA duplex (formed by chains E, F, H, and I in the Ets-2 structure) as dictated by the spacing requirements of the GGAA motifs of the Ets-2 consensus sequence using the program COOT (28). For the case of substrates with the GGAA motifs separated by 4 bp, the result of this superposition was found to be almost identical to the arrangement of the chains in the Ets-1 stromelysin-1 promoter DNA complex (20).

## RESULTS

### 

#### 

##### Structure of the Ets-2 DNA Complex

**FIGURE 1.**
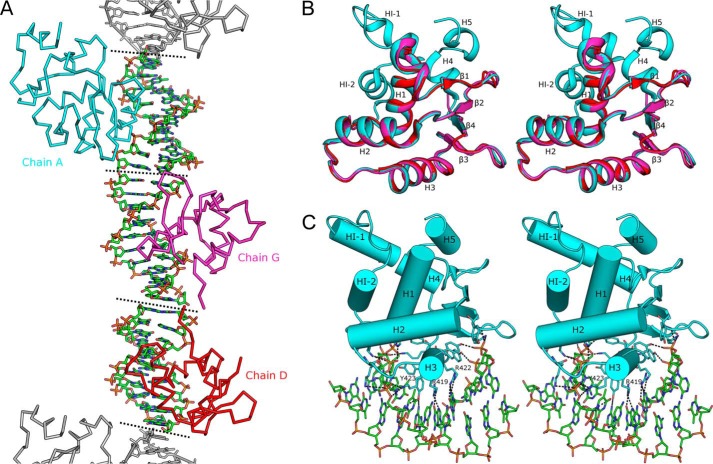
**Structure of the Ets-2 Ets bound to DNA.** *A*, schematic representation of the contents of the asymmetric unit of Ets-2; DNA crystals with Ets-2 molecules represented in the ribbon format. Crystallographic symmetry mates are shown in *gray*, and *dotted lines* represent stacking of blunt ends of DNA oligonucleotides, which form a pseudo-continuous DNA molecule in the crystal. *B*, stereo view of the overall structure of the Ets-2 Ets domain with secondary structure elements labeled. The three chains are superposed and color-coded as for *A. C*, stereo view of the interaction of Ets-2 with DNA with key interacting residues and DNA molecules shown in the stick format.

Crystals of the Ets-2 DNA complex were obtained with an Ets-2 construct spanning residues 325–464, including the N-terminal (HI-1 and HI-2) and C-terminal (H4 and H5) autoinhibitory regions and the DNA oligonucleotides 5′-ACCGGAAGTG and 5′-CACTTCCGGT. The crystals belong to a primitive monoclinic crystal system, space group P 2_1_, and diffracted to 2.5 Å resolution with 3 copies of Ets-2 and three 10-bp DNA duplexes in the asymmetric unit. The structure was solved by molecular replacement using the structure of FEV DNA complex (PDB ID 3ZP5) as a search model. The three DNA duplexes in the asymmetric unit pack with base-stacking interactions that are extended through contacts with symmetry mates to form a pseudo-contiguous double helix spanning the length of the crystal (Fig. 1*A*). The electron density map is of overall high quality for both DNA and protein chains, with the final model containing 301 of a possible 417 protein residues, and was refined to a crystallographic R_factor_ of 0.235 (R_free_ of 0.255). A summary of the data collection and refinement statistics can be found in Table 1.

**TABLE 1:** **Data collection, phasing, and refinement statistics** Data are provided for the ETS DNA complex.

**Data collection statistics**	
Space group	P 2_1_
Cell dimensions, *a*, *b*, *c* (Å)	36.7, 97.0, 83.8
Angles α, β, γ (°)	90, 97.1, 90
Wavelength (Å)	0.9765
Resolution (Å)	48-2.5 (2.62-2.5)
R_merge_*^a^*	0.04 (0.67)
R_p.i.m._*^b^*	0.03 (0.53)
*I*/σ*I*	16.3 (2.0)
Completeness (%)	99.6 (99.9)
Multiplicity	3.8 (3.9)
No. unique reflections	20,136 (2707)

**Refinement statistics**	
Resolution	48-2.5 (2.56-2.5)
R_work_/R_free_ (%)	23.4/25.6 (32.5/33.8)
No. atoms	
Protein	2,459
DNA	1,212
Ligand/ion	0
Waters	33
Average B factors (Å^2^)	
All atoms	67.9
Protein	78.5
Ligand/ion	
DNA	63.7
Waters	53.2
Wilson B	63.4
Root mean square deviations	
Bond lengths (Å)	0.003
Bond angles (°)	0.79
Ramachandran plot	
Favored (%)	97
Allowed (%)	100
PDB ID	4BQA

*^a^* R_merge_ = Σ*_hkl_*Σ*_i_*|*I_i_* − *I_m_*|/Σ*_hkl_*Σ*_i_I_i_* where *I_i_* and *I_m_* are the observed intensity and mean intensity of related reflections, respectively.

*^b^* R_p.i.m._ = Σ*_hkl_* √(1/*n* − 1) Σ_*i*_^*n*^ = 1|*I_i_* − *I_m_*|/Σ*_hkl_*Σ*_i_I_i_*.

An examination of the three Ets-2 molecules in the asymmetric unit reveals that, although they all share the same core Ets family fold and interact with double-stranded DNA in a similar manner, the autoinhibitory regions are significantly different, being well ordered in chain A but almost completely disordered in the other two chains (*D* and *G*) (Fig. 1*B*). The core Ets family fold, common to all chains in the asymmetric unit, consists of a four-stranded antiparallel β-sheet, (strand order β1, β2, β4, β3) flanked on one side by three α-helices with an additional short helix immediately preceding the first β strand, which is of intermediate character (α and 3_10_). The recognition helix (H3) inserts deep into the major groove of the DNA and forms a number of base pair-specific interactions with the DNA, with additional contacts to the DNA backbone being formed from residues in the C terminus of H2, the H2-H3 loop, β3, and the β3-β4 loop (Fig. 1*C*). Overall the protein DNA interface is very similar to that described in other structural investigations of Ets family proteins (12) and as such is not described in detail here.

##### Structure of the Autoinhibited Form

**FIGURE 2.**
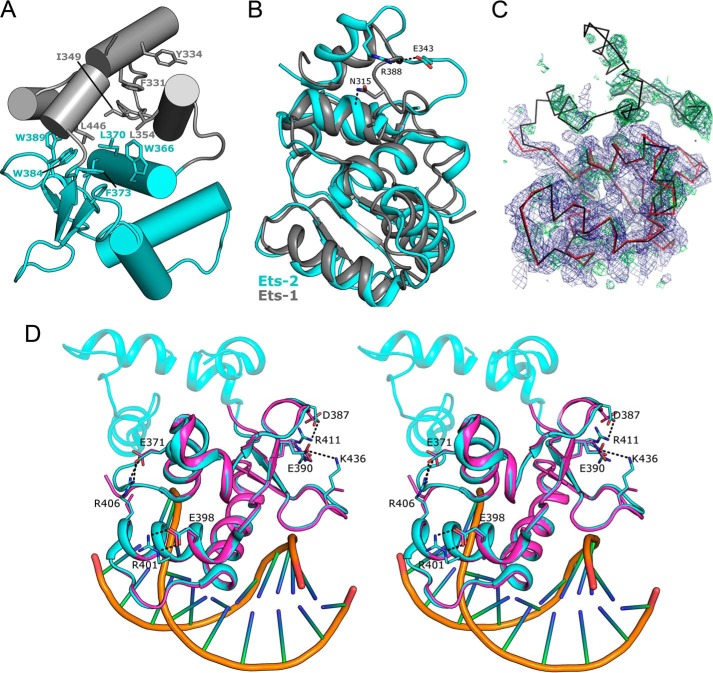
**Structure of the autoinhibited form of Ets-2.** *A*, schematic model of the interface between the core Ets domain (*cyan*) and the autoinhibitory module (*gray*). Key hydrophobic interacting residues are labeled and shown in the stick format. *B*, comparison of Ets-2 (Chain A, *cyan*) with the autoinhibited form of Ets-1 (shown in *gray*), with residues possibly contributing to the conformational difference of the HI-1-HI-2 loop shown in the stick format. *C*, 2*F_o_* − 1*F_c_* (shown in *blue*) and *F_o_* − *F_c_* (shown in *green*) electron density maps contoured at 1.0 and 2.5 σ, respectively, showing the partial electron density for the autoinhibitory module in the vicinity of chain D (shown in *red*). A copy of chain A is shown superposed for reference. *D*, stereo view of a comparison of the three Ets-2 chains in the asymmetric unit shown in the schematic representation with key residues forming intramolecular salt bridges in chain A but not in the other two chains highlighted in the stick format.

In chain A the entire autoinhibitory module can be seen to form a complete folded helical structure and associates with the core of the Ets domain via an extensive interface formed by H1 and the β1-β2 loop. The contact area for the interface is ∼790 Å^2^ with 7 hydrogen bonds being formed between the core and autoinhibitory module, although the majority of the contacts are hydrophobic in nature, with the core Ets domain providing several aromatic residues (Trp-366, Leu-370, Leu-373, Trp-384, Trp-389, and Phe-442) that contact predominantly branched chain amino acids on the autoinhibitory module (Ile-349, Leu-354, Leu-446, and Leu-457) (Fig. 2*A*). The HI-1 helix, which was suggested to undergo a structural transition from ordered to disordered upon DNA binding (8, 9), is well ordered and forms a helical secondary structure that packs against the C-terminal ends of helices H4 and HI-2, burying two predominant aromatic residues (Phe-331 and Tyr-334). Overall the structure of chain A, hereinafter referred to as the autoinhibited form, is generally similar to the NMR structure of the autoinhibited Ets-1 (7) (PDB ID 1R36) (1.7 Å root mean square deviation over 123 aligned Cα atoms). The most notable difference is the HI-1–HI-2 loop (residues 338–346), which in Ets-2 contains a single amino acid insertion (Pro-341), and is in a different conformation, forming an extended solvent-exposed loop with a single salt bridge formed between Arg-338 and Glu-343. In contrast, the equivalent region of Ets-1 folds back, forming interactions with the N-terminal region of HI-2 (Fig. 2*B*). It is interesting to note that the negatively charged Glu-343 is not conserved in Ets-1 (the equivalent residue being Asn-315) and, instead of forming a salt bridge, forms a hydrogen bond with a main chain residue within HI-2 (Ala-324). Other smaller differences can be observed in the relative positioning of HI-1, which is shifted by ∼20°, and the conformation of the β3-β4 loop, although the latter makes a number of contacts to the phosphodiester backbone of the DNA in the Ets-2 structure.

##### Structure of the Non-autoinhibited Form

**FIGURE 3.**
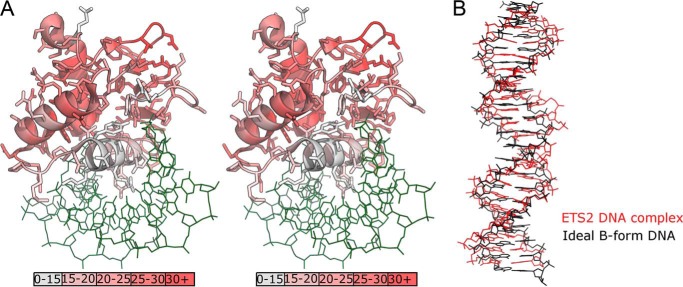
*A*, stereo view of the Ets-2 Ets domain in complex with DNA, showing the differences in B-factor between chain A and chains D and G (average of the two values used for calculation) plotted on a per-residue basis onto the structure. *B*, comparison of the geometry of the pseudo-continuous DNA strand, formed by the interface of the blunt ends of DNA molecules bound to chains D and G in the Ets-2 structure, with a similar length of ideal B-form DNA.

For the remaining chains in the asymmetric unit (chain D and G) the entire autoinhibitory module was either completely or partially disordered and was not modeled in final structure. Residual electron density, of insufficient quality to build into, can be observed in regions that would correspond to the expected positions of H1–2, H4, and H5 in chain D (Fig. 2*C*), suggesting that the disorder in this chain is less extensive. The remainder of the protein corresponding to the Ets core (residues 361–444) is overall highly similar to that of the autoinhibited form (∼0.6 Å root mean square deviation >85 aligned Cα residues), although there are some regions of significant difference between the two forms. Most notably the H1-β1 and the β1-β2 loops, which in the autoinhibited form contact the HI-1 and HI-2 loop and H5, respectively, adopt different main chain conformations. There are also a large number of side-chain residues that transition from being ordered in the autoinhibited form to being disordered in the other two chains; a significant proportion of these (Asp-387, Arg-401, Arg-406, Lys-436, and Arg-441) participate in intramolecular salt bridges in the autoinhibited form (Fig. 2*D*). Although distant from the recognition helix (H3), the even distribution of these residues around the molecule together with the fact that they generally link distant elements of secondary structure suggest that the formation of these interactions may be a way in which the presence/absence of the autoinhibitory module is transmitted allosterically. Comparing the crystallographic B factor values for the conserved regions of chains A, D, and G reveal a general pattern of significantly higher B values for regions contacting the autoinhibitory module in chains D and G, whereas the recognition helix is relatively unchanged (Fig. 3*A*).

An examination of the environment of the various chains in the asymmetric unit provides an explanation as to why two different forms are present. Although chain A does not form any significant interfaces with other Ets-2 molecules, chains D and G are closely associated, with each other being located on adjacent major grooves on the same face of the DNA in a manner highly reminiscent of other dimeric helix turn helix transcription factors. The two DNA molecules that form part of this interface stack their blunt ends together with almost exactly the same geometry as a continuous DNA double helix (Fig. 3*B*). Thus the arrangement of chains D and G in the crystal is very close to what would be expected from a palindromic DNA sequence in which the Ets binding sites are separated by six bases.

##### Autoinhibition Properties of Ets-2

Given the fact that we observe the autoinhibited form of Ets-2 bound to the specific recognition sequence in the Ets-2 structure, we decided to investigate the autoinhibitory properties of Ets-2 and compare this activity with Ets-1. We have measured the binding affinity of three different length Ets-2 constructs on a single site Ets-2 consensus DNA binding sequence directly alongside the equivalent constructs of Ets-1 (for technical reasons we have used the murine Ets-1 gene, which differs from the human gene in only one position, S288Y, over the length of the constructs used). These constructs correspond to the equivalent of viral Ets-1 (lacking the entire N-terminal autoinhibitory module), the construct used for Ets-2 crystallization, and a longer construct containing an additional 20 residues N-terminal to HI-1 that are believed to be disordered and contain a serine-rich sequence. First, the overall binding affinity of the shortest constructs, which should not be subject to any kind of autoinhibition, is significantly higher for Ets-1 than Ets-2, with an ∼4-fold difference in affinity when tested on the same substrate (0.1 ± 0.06 nm *versus* 0.4 ± 0.04 nm). Given the two proteins share ∼90% sequence identity over this region and none of the substitutions could be expected to have any direct effect on DNA binding from the crystal structure, these differences indicate that some subtle aspects of the energetics of the interaction of Ets-1 and Ets-2 with DNA are not understood.

**FIGURE 4.**
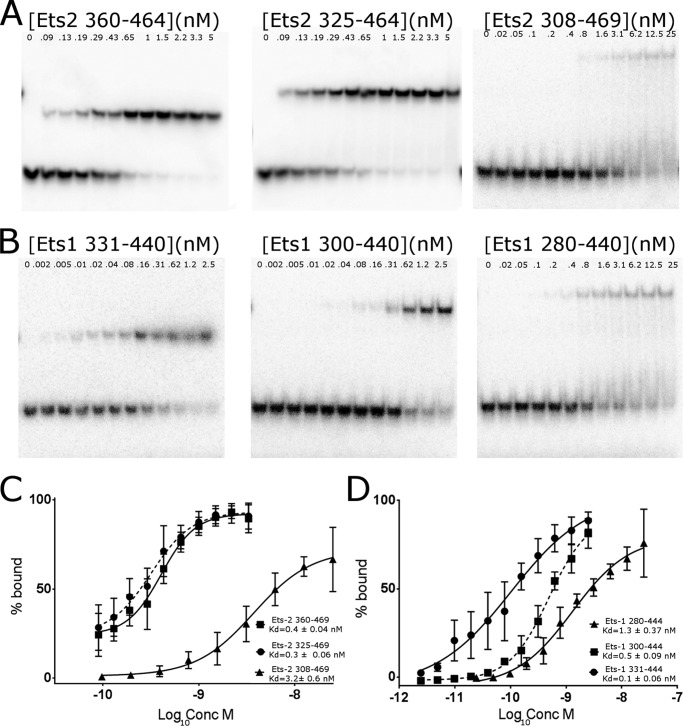
**Analysis of the autoinhibition of Ets-2.** *A*, representative electrophoretic mobility shift assays of three Ets-2 constructs binding to DNA substrates with a single copy of the consensus Ets-2 binding sequence. The constructs from *left* to *right* correspond to Ets-2^360–464^ (core Ets domain only), Ets-2^325–464^ (core Ets and autoinhibition domains), and Ets-2^308–469^ (core, autoinhibition and N-terminal serine-rich sequence). *B*, representative electrophoretic mobility shift assays of the equivalent domains of Ets-1 with the same DNA sequence. *C*, quantification of the data presented in *panel A*; *error bars* are plotted as ±S.E. from at least three independent experiments, and the data are plotted for *K*_*d*_^app^. *D*, quantification of the Ets-1 binding data presented in *panel B*.

We have also found significant differences between Ets-1 and Ets-2 in the nature of their autoinhibition. In Ets-1 two mechanisms of autoinhibition have been found to be present, a modest effect caused by the association of the autoinhibitory module (2–3-fold inhibition) (4–6, 8, 9) and a more marked inhibition provided by an intrinsically disordered serine-rich sequence N-terminal to this (13–15). Consistent with previous studies, we were able to demonstrate both types of inhibition on Ets-1 (∼5- and 2.6-fold, respectively), but only the latter mechanism of inhibition seems to apply to Ets-2 (∼8-fold inhibition; Fig. 4).

##### Cooperative DNA Binding by Ets-2

Cooperative binding to palindromic EBS repeats has been the focus of numerous studies using both Ets-2 and Ets-1 (9, 16, 20, 21), with the majority of studies focusing on palindromic arrangements such as that in the stromelysin promoter in which the core GGA(A) motif is separated by four base pairs. Given the differences observed in the autoinhibition between Ets-1 and Ets-2 and the finding of an arrangement in the crystal structure that mimics a palindromic arrangement separated by 6 bp, we have decided to perform electrophoretic mobility shift assays using the short and medium length Ets-2 constructs. These were incubated with a variety of DNA substrates containing inverted repeats of the consensus Ets binding motif separated by a variable 4–8-bp spacer.

**FIGURE 5.**
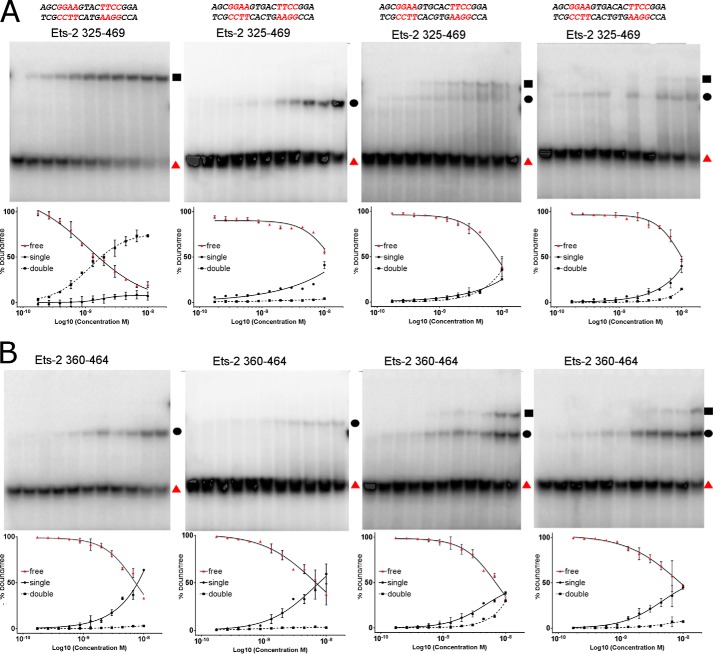
**Analysis of cooperative DNA binding by Ets-2.** *A*, cooperative DNA binding properties of Ets-2^325–464^ (core Ets and autoinhibition domains) binding to a variety of DNA substrates with palindromic repeats, with a variable length spacer between the GGAA core. DNA sequences used are shown on the *top*, representative electrophoretic mobility shift assays are shown in the *center*, and a quantification plot, which expresses the amounts of free DNA (*red triangles*), single-shifted species (*black circles*), and double-shifted species (*black squares*) as a percentage of the total material in each lane, is shown on the *bottom. Error bars* are plotted as ±S.E. from two independent experiments, although additional biological replicates were performed with the same results. *B*, cooperative DNA binding properties of Ets-2^360–464^ (core Ets domain only). The data are presented as for *A* with the DNA substrate used being the same as that used above.

The binding of Ets-2 to substrates with a palindromic arrangement was different between the two constructs, with the Ets-2 325–469 construct binding exclusively in a cooperative manner (forming a super-shifted band on the gel, which we presume represents an Ets-2 DNA complex with 2:1 stoichiometry) to substrates with a 4-bp spacer, and exclusively in a non-cooperative manner to substrates with a 5-bp spacer (Fig. 5*A*). Substrates containing 6- or 7-bp spacers appeared to be able to form both single and super-shifted species with both constructs (Fig. 5*A*). We performed a crude quantification of these data, taking the disappearance of the free DNA as a proxy for the total binding, and the appearance of single- and double-shifted bands to represent 1:1 and 2:1 interaction stoichiometries (Fig. 5, *A* and *B*). Although the data do not reach saturation it is clear that some degree of cooperativity is in effect in the binding of Ets-2 to the 6-bp substrate, which clearly shows significant amounts of the double-shifted band at lower protein concentrations than the 7-bp substrate (which we infer to be independent binding events). Data for substrates with 8-bp spacer sequences were found to be almost identical to the 7-bp data and for the sake of brevity are not shown. The extent of the cooperativity on the 6-bp substrate is significantly less marked than observed with the 4-bp substrate, and the fact that it is observed (although possibly to different extents) in both constructs suggests it is not dependent on residues in the autoinhibitory region. It is important to note, however, that in all cases the apparent affinity for the palindromic sequences is significantly lower than for the single-site substrate, perhaps indicating a sequence dependence on DNA binding.

**FIGURE 6.**
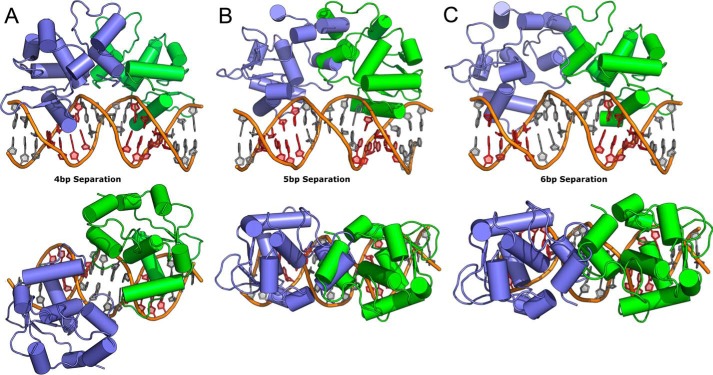
**Modeling of the Ets-2 structures on to three DNA substrates in which the core GGA(A/T) motif is separated by 4 (*A*), 5 (*B*), and 6 base pairs (*C*) viewed with the 2-fold symmetry axis vertical (*upper row*) and in the plane on the page (*lower row*).**

Using the DNA-bound structures of Ets-2 as a guide it is possible to model the potential intermolecular interactions formed on these substrates. In agreement with observations from crystal structures of Ets-1 in complex with the stromelysin promoter DNA (19, 20), positioning two copies of Ets-2 on substrates with a 4-bp spacer reveals a small but significant protein interface area (∼370 Å^2^) created by the HI-2-H1 loop contacting the H2-H3 loop in the adjacent chain, with the potential to form the same hydrogen bond between Asn-408 and Gly-361 as found in the structure of Ets-1 bound to the stromelysin promoter (19, 20) (Fig. 6*A*). Crucially this interface is unlikely to be formed in the shorter construct which lacks HI-1 and HI-2. Positioning Ets-2 on substrates with a 5-bp separation reveals significant steric clashes that occur between the two molecules involving primarily HI-1 and HI-2 but also regions of the core Ets domain (Fig. 6*B*), explaining the complete lack of formation of the super-shifted band in either construct. The likely arrangement of Ets-2 on a substrate with binding sites separated by 6 bp is revealed by chains D and G in the Ets-2 structure, and positioning two copies of the autoinhibited form onto these chains reveals a steric clash occurs between the C-terminal ends of HI-1 and HI-2 and their equivalents on the neighboring subunit (Fig. 6*C*). Nevertheless the fact that this arrangement is present in the Ets-2 crystals (which were obtained using the longer Ets-2 325–469 construct) and appears, from the EMSA analysis, to form preferentially in the longer construct but not the short, suggests that this clash can be accommodated presumably by the introduction of disorder in the N-terminal inhibitory module. Thus the structure suggests that there is a direct steric requirement for at least one of the autoinhibitory modules to be disordered when binding to this type of substrate.

**FIGURE 7.**
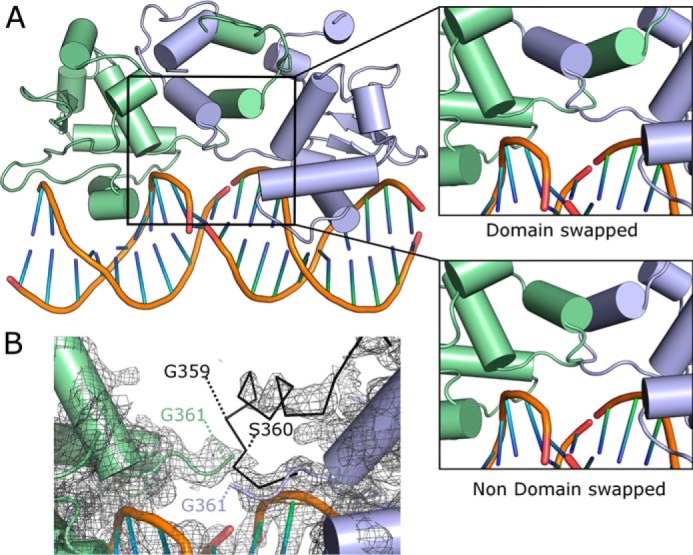
**Possible domain swapping in Ets-2.** *A*, superposition of Ets-2 Ets domain chain A onto chains D and G reveals the potential for domain swapping of the entire N-terminal autoinhibitory module to occur. *B*, view of the 2*F_o_* − 1*F_c_* electron density map in this region contoured at 1.0 σ. The *black ribbon diagram* represents the path of the molecule for the non-domain-swapped species formed by superposition of chain A onto chain D.

We have also noticed through this superposition that, due to the fact that equivalent regions of the HI-2-H1 loop overlap close to residues Gly-361 and Pro-362, it is possible that the N-terminal autoinhibitory modules may adopt a domain-swapped conformation (Fig. 7*A*). Although the electron density is too poor to determine whether this is happening in the crystal (Fig. 7*B*), the fact that numerous examples of domain swapping have also occurred in various Ets-1 structures suggests that this phenomenon may be worthy of a more detailed investigation. Modeling of Ets-2 structures bound to substrates with Ets binding sites separated by seven or more base pairs reveals that there is no possibility of forming a significant protein-protein interface, and thus the binding at the two sites would be expected to be largely independent.

## DISCUSSION

Previous structural studies of the closely related Ets-1 established a model for autoinhibition in which the unfolding of HI-1 upon binding DNA (a process that presumably has a positive Δ*G*) reduces the affinity of the autoinhibited form. It had been presumed that this mechanism may also apply to Ets-2; however, we failed to demonstrate any noticeable difference in DNA binding affinity on single Ets binding sites between Ets domain constructs with and without the N-terminal autoinhibitory regions. In contrast we have shown an ∼5-fold reduction in affinity when comparing the equivalent constructs of Ets-1, which is in agreement with previous studies (4–6, 8, 9, 30). Comparison of the two DNA complex structures reveals an almost identical protein DNA interface, with the sequence of the recognition helix and all DNA contacting residues being completely conserved between the two proteins, indicating that the differences in affinity may come from the dynamics of the association of the autoinhibitory module. Instead the serine-rich region upstream of the N-terminal autoinhibitory region, which has been shown to play a role in Ets-1 inhibition (10, 13, 15), appears to be the sole region responsible for the autoinhibition of Ets-2, causing an ∼10-fold inhibition. It is possible that this inhibition may be further enhanced by phosphorylation as is the case for Ets-1 (15).

We were also surprised to find two distinct forms for the Ets domain of Ets-2 in our crystals, one in which the N-terminal autoinhibitory regions are largely disordered and another with the entire autoinhibitory region ordered. The fact that both of these are bound to DNA and appear to make an identical protein DNA interface again suggests that the established model of Ets-1 autoinhibition is not directly applicable to Ets-2. Nevertheless a comparison of the two forms of Ets-2 gives insights into how the presence or absence of the autoinhibitory module may be transmitted to the rest of the Ets domain, with a number of intramolecular salt bridges formed in the autoinhibited form (presumably stabilized by the presence of the autoinhibitory module) which are evenly distributed throughout the molecule and may serve to subtly alter the conformation or dynamics of the residues forming the DNA interface.

We have also investigated the cooperativity of DNA binding by Ets-2 and in this respect find the activity of Ets-2 to be largely similar to Ets-1. A significant cooperative DNA binding effect was observed on DNA substrates where the Ets binding sites were separated by 4 bp, which was dependent on the presence of the N-terminal autoinhibitory helices. The length dependence of the DNA spacer was also investigated, and it was found that Ets-2 was unable to bind cooperatively to sites separated by 5 bp. Some degree of cooperativity in binding was observed for sites separated by 6 bp, which in contrast to the situation in the 4-bp-spaced sequences was effected by, but not dependent on the presence of the autoinhibitory region. Modeling of Ets-2 onto these various substrates revealed the possible mechanism by which cooperativity is conferred due to the potential to form favorable/unfavorable interactions and order-disorder transitions of the N-terminal autoinhibitory helices.

### 

#### 

##### Protein Data Bank Accession Numbers

Atomic coordinates and structure factors were deposited in the Protein Data Bank with the accession number PDB 4BQA (Ets-2-DNA).

## Footnotes

4 The abbreviations used are:

Ets: E26 transformation-specific
EBS: Ets binding site
BisTris: 2-[bis(2-hydroxyethyl)amino]-2-(hydroxymethyl)propane-1,3-diol.

## References

[1] Degnan B. M. Degnan S. M. Naganuma T. Morse D. E. (1993) The ets multigene family is conserved throughout the Metazoa. Nucleic Acids Res. 21, 3479–3484 8346026 10.1093/nar/21.15.3479 PMC331448

[2] Wang Z. Zhang Q. (2009) Genome-wide identification and evolutionary analysis of the animal specific ETS transcription factor family. Evol. Bioinform. Online 5, 119–131 20011068 10.4137/ebo.s2948 PMC2789578

[3] Wei G. H. Badis G. Berger M. F. Kivioja T. Palin K. Enge M. Bonke M. Jolma A. Varjosalo M. Gehrke A. R. Yan J. Talukder S. Turunen M. Taipale M. Stunnenberg H. G. Ukkonen E. Hughes T. R. Bulyk M. L. Taipale J. (2010) Genome-wide analysis of ETS-family DNA binding *in vitro* and *in vivo*. EMBO J. 29, 2147–2160 20517297 10.1038/emboj.2010.106 PMC2905244

[4] Hagman J. Grosschedl R. (1992) An inhibitory carboxyl-terminal domain in Ets-1 and Ets-2 mediates differential binding of ETS family factors to promoter sequences of the mb-1 gene. Proc. Natl. Acad. Sci. U.S.A. 89, 8889–8893 1409581 10.1073/pnas.89.19.8889 PMC50029

[5] Lim F. Kraut N. Framptom J. Graf T. (1992) DNA binding by c-Ets-1, but not v-Ets, is repressed by an intramolecular mechanism. EMBO J. 11, 643–652 1311254 10.1002/j.1460-2075.1992.tb05096.x PMC556496

[6] Wasylyk C. Kerckaert J. P. Wasylyk B. (1992) A novel modulator domain of Ets transcription factors. Genes Dev. 6, 965–974 1592263 10.1101/gad.6.6.965

[7] Lee G. M. Donaldson L. W. Pufall M. A. Kang H. S. Pot I. Graves B. J. McIntosh L. P. (2005) The structural and dynamic basis of Ets-1 DNA binding autoinhibition. J. Biol. Chem. 280, 7088–7099 15591056 10.1074/jbc.M410722200

[8] Petersen J. M. Skalicky J. J. Donaldson L. W. McIntosh L. P. Alber T. Graves B. J. (1995) Modulation of transcription factor Ets-1 DNA binding: DNA-induced unfolding of an α helix. Science 269, 1866–1869 7569926 10.1126/science.7569926

[9] Jonsen M. D. Petersen J. M. Xu Q. P. Graves B. J. (1996) Characterization of the cooperative function of inhibitory sequences in Ets-1. Mol. Cell. Biol. 16, 2065–2073 8628272 10.1128/mcb.16.5.2065 PMC231193

[10] Cowley D. O. Graves B. J. (2000) Phosphorylation represses Ets-1 DNA binding by reinforcing autoinhibition. Genes Dev. 14, 366–376 10673508 PMC316366

[11] Goetz T. L. Gu T. L. Speck N. A. Graves B. J. (2000) Autoinhibition of Ets-1 is counteracted by DNA binding cooperativity with core-binding factor α2. Mol. Cell. Biol. 20, 81–90 10594011 10.1128/mcb.20.1.81-90.2000 PMC85055

[12] Garvie C. W. Pufall M. A. Graves B. J. Wolberger C. (2002) Structural analysis of the autoinhibition of Ets-1 and its role in protein partnerships. J. Biol. Chem. 277, 45529–45536 12221090 10.1074/jbc.M206327200

[13] Lee G. M. Pufall M. A. Meeker C. A. Kang H. S. Graves B. J. McIntosh L. P. (2008) The affinity of Ets-1 for DNA is modulated by phosphorylation through transient interactions of an unstructured region. J. Mol. Biol. 382, 1014–1030 18692067 10.1016/j.jmb.2008.07.064 PMC4808631

[14] Pufall M. A. Lee G. M. Nelson M. L. Kang H. S. Velyvis A. Kay L. E. McIntosh L. P. Graves B. J. (2005) Variable control of Ets-1 DNA binding by multiple phosphates in an unstructured region. Science 309, 142–145 15994560 10.1126/science.1111915

[15] Desjardins G. Meeker C. A. Bhachech N. Currie S. L. Okon M. Graves B. J. McIntosh L. P. (2014) Synergy of aromatic residues and phosphoserines within the intrinsically disordered DNA-binding inhibitory elements of the Ets-1 transcription factor. Proc. Natl. Acad. Sci. U.S.A. 111, 11019–11024 25024220 10.1073/pnas.1401891111 PMC4121781

[16] Venanzoni M. C. Robinson L. R. Hodge D. R. Kola I. Seth A. (1996) ETS1 and ETS2 in p53 regulation: spatial separation of ETS binding sites (EBS) modulate protein:DNA interaction. Oncogene 12, 1199–1204 8649821

[17] Baillat D. Bègue A. Stéhelin D. Aumercier M. (2002) ETS-1 transcription factor binds cooperatively to the palindromic head to head ETS-binding sites of the stromelysin-1 promoter by counteracting autoinhibition. J. Biol. Chem. 277, 29386–29398 12034715 10.1074/jbc.M200088200

[18] Baillat D. Leprivier G. Régnier D. Vintonenko N. Bègue A. Stéhelin D. Aumercier M. (2006) Stromelysin-1 expression is activated *in vivo* by Ets-1 through palindromic head-to-head Ets binding sites present in the promoter. Oncogene 25, 5764–5776 16652151 10.1038/sj.onc.1209583

[19] Lamber E. P. Vanhille L. Textor L. C. Kachalova G. S. Sieweke M. H. Wilmanns M. (2008) Regulation of the transcription factor Ets-1 by DNA-mediated homo-dimerization. EMBO J. 27, 2006–2017 18566588 10.1038/emboj.2008.117 PMC2486274

[20] Babayeva N. D. Wilder P. J. Shiina M. Mino K. Desler M. Ogata K. Rizzino A. Tahirov T. H. (2010) Structural basis of Ets1 cooperative binding to palindromic sequences on stromelysin-1 promoter DNA. Cell Cycle 9, 3054–3062 20686355 10.4161/cc.9.15.12257 PMC2928650

[21] Babayeva N. D. Baranovskaya O. I. Tahirov T. H. (2012) Structural basis of Ets1 cooperative binding to widely separated sites on promoter DNA. PloS ONE 7, e33698 22432043 10.1371/journal.pone.0033698 PMC3303851

[22] Hollenhorst P. C. Jones D. A. Graves B. J. (2004) Expression profiles frame the promoter specificity dilemma of the ETS family of transcription factors. Nucleic Acids Res. 32, 5693–5702 15498926 10.1093/nar/gkh906 PMC524310

[23] Shaikhibrahim Z. Ochsenfahrt J. Fuchs K. Kristiansen G. Perner S. Wernert N. (2012) ERG is specifically associated with ETS-2 and ETV-4, but not with ETS-1, in prostate cancer. Int. J. Mol. Med. 30, 1029–1033 22922762 10.3892/ijmm.2012.1097 PMC3572757

[24] Guen V. J. Gamble C. Flajolet M. Unger S. Thollet A. Ferandin Y. Superti-Furga A. Cohen P. A. Meijer L. Colas P. (2013) CDK10/cyclin M is a protein kinase that controls ETS2 degradation and is deficient in STAR syndrome. Proc. Natl. Acad. Sci. U.S.A. 110, 19525–19530 24218572 10.1073/pnas.1306814110 PMC3845122

[25] Savitsky P. Bray J. Cooper C. D. Marsden B. D. Mahajan P. Burgess-Brown N. A. Gileadi O. (2010) High-throughput production of human proteins for crystallization: the SGC experience. J. Struct. Biol. 172, 3–13 20541610 10.1016/j.jsb.2010.06.008 PMC2938586

[26] Kabsch W. (2010) XDS. Acta Crystallogr. D Biol. Crystallogr. 66, 125–132 10.1107/S0907444909047337 PMC2815665 20124692

[27] Vagin A. Teplyakov A. (1997) MOLREP: an automated program for molecular replacement. J. Appl. Crystallogr. 10.1107/S0021889897006766

[28] Emsley P. Lohkamp B. Scott W. G. Cowtan K. (2010) Features and development of Coot. Acta Crystallogr. D Biol. Crystallogr. 66, 486–501 20383002 10.1107/S0907444910007493 PMC2852313

[29] Adams P. D. Afonine P. V. Bunkóczi G. Chen V. B. Davis I. W. Echols N. Headd J. J. Hung L. W. Kapral G. J. Grosse-Kunstleve R. W. McCoy A. J. Moriarty N. W. Oeffner R. Read R. J. Richardson D. C. Richardson J. S. Terwilliger T. C. Zwart P. H. (2010) PHENIX: a comprehensive Python-based system for macromolecular structure solution. Acta Crystallogr. D Biol. Crystallogr. 66, 213–221 20124702 10.1107/S0907444909052925 PMC2815670

[30] Wang H. McIntosh L. P. Graves B. J. (2002) Inhibitory module of Ets-1 allosterically regulates DNA binding through a dipole-facilitated phosphate contact. J. Biol. Chem. 277, 2225–2233 11689571 10.1074/jbc.M109430200

